# The Effect of Juggling as Dual-Task Activity on Human Neuroplasticity: A Systematic Review

**DOI:** 10.3390/ijerph19127102

**Published:** 2022-06-09

**Authors:** Jakub Malik, Rafał Stemplewski, Janusz Maciaszek

**Affiliations:** 1 Department of Physical Activity and Health Promotion Science, Poznan University of Physical Education, Królowej Jadwigi 27/39, 61-871 Poznan, Poland; jmaciaszek@awf.poznan.pl; 2 Department of Digital Technologies in Physical Activity, Poznan University of Physical Education, Królowej Jadwigi 27/39, 61-871 Poznan, Poland; stemplewski@awf.poznan.pl

**Keywords:** bimanual task, human brain, neural plasticity

## Abstract

This systematic review formulated a research question based on the PICO method in accordance with the Guidelines for Systematic Reviews and Meta-Analyses (PRISMA), “What is the effect of juggling as dual-task activity on neuroplasticity in the human brain?” In total, 1982 studies were analysed, 11 of which met the inclusion criteria and were included in the review. These studies included 400 participants who had no prior juggling experience or were expert jugglers. The research methodology in seven studies was based on a long-term intervention with juggling. Three studies were based on brain imaging during the act of juggling, and one study was based on comparing differences between experienced jugglers and non-jugglers without the intervention. In all of these selected studies, positive structural changes in the human brain were found, including changes mainly in the gray matter (GM) volume in the visual motion complex area (hMT/V5) and the white matter (WM) volume in fractional anisotropy (FA). Based on this evidence, it can be concluded that the bimanual juggling task, as a dual-task activity, may effectively integrate brain areas to improve neuroplasticity. The small number of well-designed studies and the high risk of bias call for further research using a juggling intervention to identify conclusive evidence.

## 1. Introduction

Research interest in dual-task training continues. It is defined as the ability to perform two or more cognitive and motor activities simultaneously. The ability to divide the attention at the same time between two or more tasks is an important aspect of functional movement during daily activities [1]. During these activities, people not only stabilize their body posture at all times but also simultaneously undertake the continuous performance of other cognitive activities or motor tasks. A dual-task training protocol usually consists of a primary motor task (e.g., walking or balancing) and a secondary task that requires attention (e.g., a motor or cognitive task) [2]. It may cause great concern in situations of fatigue after exercise where postural control is decreased, especially in seniors with a lower level of habitual physical activity [3].

Daily activities do not require deliberating on the complexity of the movements the body performs. The usage of both hands to manipulate an object or to perform a specific task is common. However, most people have a dominant hand that is more efficient at making precise movements, development of which begins in childhood and continues almost throughout the entire life. Many of the tools that are used in everyday life are designed for right-handed people. Thus, the non-dominant side of the body is often neglected [4,5].

Juggling, as a sensorimotor task requiring complex visuomotor control and interaction between both limbs, may enhance the bilateral transfer learning effect that has been observed for the upper and lower limbs from non-dominant limb training [6,7,8]. This effect shows that the efficiency of one side of the body can be increased by developing the other side of the body [9]. Moreover, classical juggling, as an activity involving simultaneously throwing and catching balls in a specific motor pattern with both hands and paying attention to the trajectory of each ball without hesitation, can be considered a dual-task motor activity. It has been shown that individuals who juggle professionally achieve lower swing amplitude during stabilometric measurements when simultaneously performing a three-ball cascade. More experienced jugglers not only perform the task more accurately but also become more automatic. As a result, the cognitive resources of attention of professional jugglers are not as exploited by juggling as in the case of beginner jugglers, whose swing amplitude during juggling is higher [10].

The currently available in vivo imaging techniques of human brain structures have revealed selective activity-dependent changes in the adult brain structure [11]. This observation is strongly supported by the current evidence, confirming that physical exercise has beneficial effects on neuroplasticity and can also improve human cognition [11,12,13,14]. More importantly, learning new motor skills brings about changes in regional brain morphology. For example, one study found an increase in the volume of the parahippocampal region, as well as the GM in the left precentral cortex after an 18-month dance intervention in older adults [15]. Next, Kattenstroth et al. [16] showed that moderate sessions of physical activity that are not sufficient to affect cardiorespiratory fitness but are sufficiently cognitively challenging can have beneficial effects on cognition, posture, balance, and sensorimotor performance. According to these findings, it might be concluded that participation in an activity that requires continuous cognitive and motor learning provides greater neuroplasticity benefits than repetitive physical exercise [15]. Furthermore, it is claimed that learning bilateral motor tasks may result in greater engagement of the cerebral hemispheres, depending on the stage of learning [17].

Learning to juggle as a new movement task can be an interesting form of bilateral activity which, due to its complexity, may have a positive influence on neuroplasticity in people of all ages. What is important, in order to practice juggling, there is no need for specific and expensive equipment or spacious surroundings. The very act of juggling seems to be a safe activity for people of all at every stage of life [18].

Therefore, the purpose of this review was to determine how classical juggling, as a dual-task activity, affects neuroplasticity in the human brain.

## 2. Methods

### 2.1. Search Strategies

The review was conducted according to PRISMA (Preferred Reporting Items for Systematic Reviews and Meta-analysis) guidelines and followed the recommendations of the international PRESS (Peer Review of Electronic Search Strategies) guidelines. The 27-item PRISMA 2020 checklist was used in the development of the systematic review. The systematic review protocol was registered with PROSPERO (International Prospective Register of Systematic Reviews) under the number CRD42021272053. Sources were searched for electronically in four literature databases (PubMed, Web of Science, EBSCOhost, and Scopus). The last search for sources was performed in December 2021. The search was based on the following index terms: ((‘juggl* [all fields]) AND (‘neuro*’ [all fields] OR ‘plasticity’ [all fields] OR ‘brain’ [all fields])), where ‘*’ indicates any other ending of the assigned word. The search was also supplemented with an Internet search (Google Scholar), as well as forward and backward citation tracking from systematic reviews and the included studies.

### 2.2. Inclusion and Exclusion Criteria

Original articles written in English were included in the article search, without restriction as to the date of publication. All studies conducted on older adults, adults or children, men or women, healthy or not, physically active or not, and who were subjected to a juggling intervention with neuroplasticity effects were included. Exclusion criteria included studies conducted on animals, and studies conducted with non-classical juggling (leg juggling, contact juggling, and partner juggling were excluded).

### 2.3. Data Extraction

One researcher was responsible for data extraction and evaluation. The data extraction was checked independently by two other authors. In the first step, a list of all studies was extracted; in the next step, all duplicates were removed; in the third step, a selection of the titles and abstracts that were identified as potentially eligible studies was made; in the final step, the full texts of the articles were evaluated for eligibility. Each selected publication was subjected to critical analysis.

### 2.4. Quality Assessment of the Experiments

**Figure 1:**
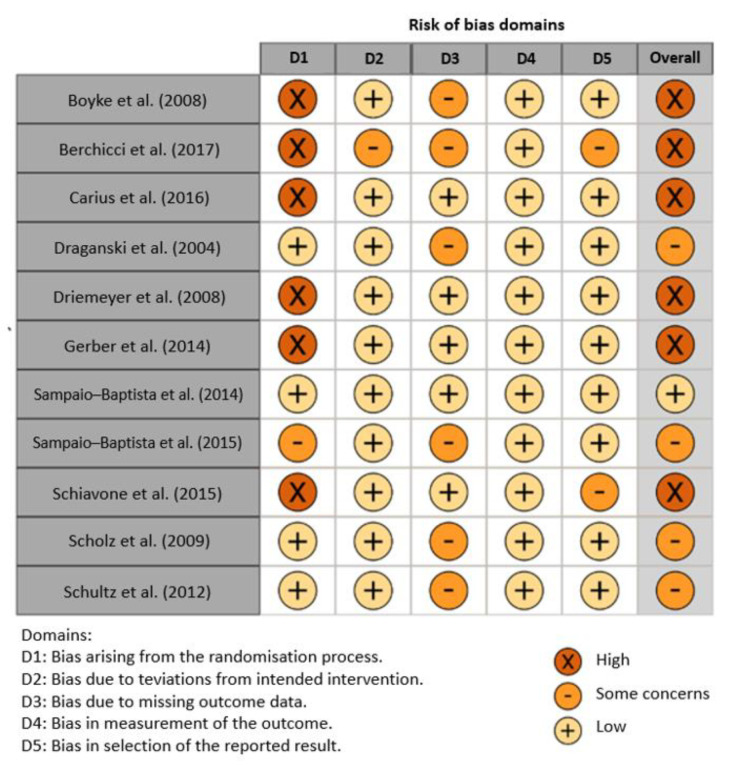
Risk of bias of all included studies [20,21,22,23,24,25,26,27,28,29,30].

**Figure 2:**
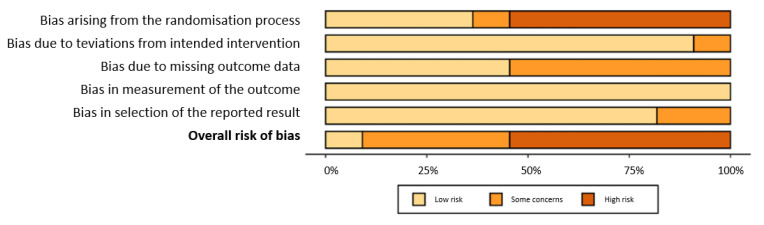
Risk of bias summary: reviewing authors’ judgements about each risk of bias item for each included study.

The risk of bias was independently assessed by two researchers using the latest version of the Cochrane Collaboration Risk-of-Bias tool (RoB 2.0 data) [19] for randomised trials. The tool includes algorithms that give a proposed risk-of-bias score for each domain at three levels: low, some concern, and high. All studies were assessed in five domains: bias due to the randomisation process, bias due to deviations from the intended intervention, bias due to missing outcome data, bias in outcome measurements, and bias in selection of the reported outcome. The main area with the highest risk of bias was the randomisation process. Six of the included studies had a high risk of error in this domain, one had some risk, and five had a low risk. As a result, six studies were rated as having a high risk of error, four as having an unclear risk of error, and one as being low-risk. The details are shown in Figure 1 and Figure 2.

## 3. Results

### 3.1. Main Search

**Figure 3:**
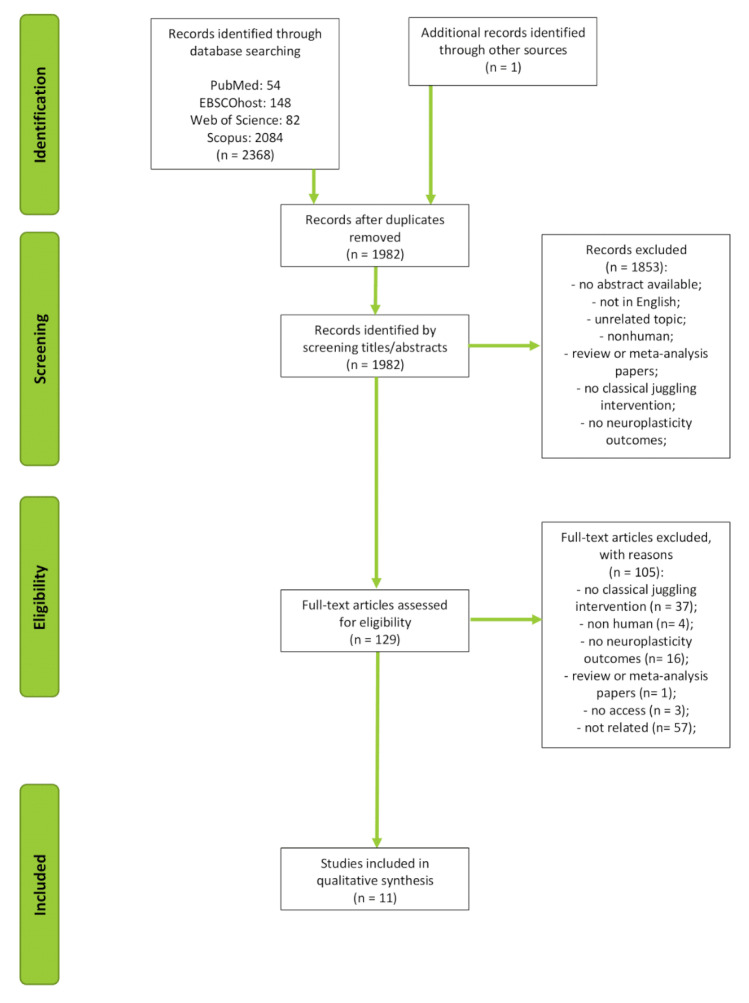
Study selection process.

In the electronic search, 2368 potential studies in the databases were identified (PubMed: *n* = 54, EBSCOhost: *n* = 148, Web of Science: *n* = 82; Scopus: *n* = 2084). One additional article was identified from other sources (Google Scholar). After removing 387 duplicates, 1982 records were evaluated by checking the titles and abstracts against the inclusion criteria, and 1853 studies that clearly did not address juggling and neuroplasticity were excluded. In total, 129 articles were subjected to full-text review. Of these studies, 118 were unrelated to the topic. Some did not include a classical juggling intervention (*n* = 37); others did not include results on neuroplasticity (*n* = 16), were non-human studies (*n* = 4), were not available (*n* = 3), or were reviews (*n* = 1). The remaining excluded articles were found to be unrelated to the topic according to any of the above requirements (*n* = 57). Ultimately, only 11 studies were included in the final analysis. The study selection process is shown in Figure 3.

### 3.2. Study Characteristics

Eleven studies involving 400 participants were included in the study (200 men; 170 women; 30 not specified). Of these 11 studies, eight included participants who had no prior juggling experience [20,21,23,24,26,27,29,30]. Four articles included participants who had experience in juggling [21,22,25,28]. The characteristics and evaluations of the study group are shown in Table 1. The studies were published between 2004 and 2017. Seven of them were conducted in Germany [20,22,23,24,25,29,30], two in the United Kingdom [26,27], one in the Netherlands [28], and one in Italy [21]. Seven of the included studies implemented a long-term intervention [20,23,24,26,27,29,30]. Three articles described a short intervention that could be implemented within 1 day [20,28,30]. In one study, the evaluation was conducted without an intervention with a specific cohort [25]. Changes in neuroplasticity were evaluated by use of magnetic resonance imaging (MRI), electroencephalography (EEG), voxel-based morphometry (VBM), or functional near-infrared spectroscopy (fNIR). Others also used surface electromyography (sEMG), kinematic data, and other assessments that are not directly described as neurological variables. Six of the included studies were randomised controlled trials (RCTs) [21,23,26,27,29,30]. Table 2 provides a summary of the study design, procedure, intervention period and frequency, measurement time points, and outcomes. The measurements in each article did not follow the same protocol. For example, EEG studies used a sampling rate of 250 Hz [21] or 256 Hz [28]; also, the MRI data were mainly performed with a 3T MRI system [20,23,26,27,29] using the MPRAGE sequence (TR = 20.40 ms; TE = 4.7 ms; flip angle = 8°; voxel size = 1 × 1 × 1 mm^3^ [26,27]; or TR = 11.08 ms; TE = 4.0 ms; flip angle = 15°; voxel size = 0.97 × 0.97 × 1.09 mm [25]) or the 3D-FLASH sequence (TR = 15.00 ms; TE = 4.9 ms; flip angle = 25°; 1 mm slices [20,24]). VMB studies also focused on different brain regions, which did not allow for a reliable comparison among the obtained results.

## 4. Discussion

### 4.1. Summary of the Main Results

The results of this systematic review showed that juggling training appears to be a good form of activity to induce brain plasticity. All of the selected studies showed positive structural changes or differences fostered by juggling. Some of them showed that jugglers have more developed brain structures compared with non-jugglers. These differences were mainly seen in the GM density of the hMT/V5 area [25]. Moreover, other studies proved that this form of activity may develop the abovementioned brain areas and WM density in the human brain [29,30]. Furthermore, brain activity during the task was different in participants who had more experience with juggling, and these differences were observed in groups with various training intensities or task difficulty levels. As shown in one of the publications, the juggling task can be practiced safely at home without any presence of a trainer or other participants [26]. This may be a very positive aspect of this form of activity, especially in unfavorable conditions when group meetings are limited for some reason (for example, a pandemic situation). However, due to the inability to conduct a meta-analysis and the small sample sizes of the selected studies, conclusions about the effect of juggling on neuroplasticity should be approached with caution. In future studies on this topic, care should be taken to ensure an adequate sample size that provides a power of at least 0.8 to provide conclusive results regarding the effect of this physical activity on the human brain.

### 4.2. Impact of Juggling on the Brain

There are only sparse studies reporting the effects of classical juggling on neuroplasticity. The low number of studies on juggling can be explained by the inconsiderable popularity of this activity. This form of movement is commonly associated with art, which can affect the perception of juggling as a difficult skill to achieve. This misconception means that the potential of this undemanding form of activity remains untapped. Developmental research on motor skills proved that juggling can be successfully learned by children and elderly people. In this study, the performance of older people (>60 years), children (10–14 years), and older adults (30–59 years) was comparable. Only adolescents and younger adults (15–29 years) performed better than older adults. Thus, the potential for learning a novel motor task such as juggling is still high, even in older adults [18]. Moreover, during the thorough literature search, one study with an efficient juggling intervention in a group of children with spina bifida was found [31]. These promising results allow for the implementation of juggling activity regardless of the age and health ailments of a participant.

In the studies selected for this review, the intervention (if used) focused specifically on juggling. Boyke et al. [20] undertook a study of learning juggling in older adults (mean age: 60 years). The results showed that older adults retain the ability to alter their brain structure in response to motor learning needs. This study extends the knowledge of the structural changes occurring in the human brain that were observed by Draganski et al. [23], who applied a three-month juggling intervention to young adults (mean age: 22 years). Despite having the same duration of the intervention and the same instructions, the results showed a poorer final outcome of learning to juggle in the older adult group. This was also confirmed in the study of Voelcker-Rehage and Willimczik [18]. However, this difference did not translate to nervous system plasticity, as it was comparable in both studies and occurred in the same areas, resulting in a significant increase in GM in the hippocampus and nucleus accumbens and hMT/V5 in individuals practicing juggling for three months [20,23]. Additionally, Berghuis et al. [32] also showed that the motor performance of older adults (mean age: 63.1 years) was 53% lower during learning a motor task than that of young adults (mean age: 25.5 years). Based on this study, it appeared that motor practice improved motor performance similarly in both groups, while brain activation was greater in the older group. However, a difference was observed during deactivation in specific areas of the brain, depending on age. Young adults had greater deactivation from post-test to retention in the parietal, occipital, and temporal cortex, while older adults showed less deactivation in the frontal cortex. The authors concluded that this is likely due to compensatory mechanisms in older adults that activate brain areas to a greater extent during motor tasks.

Changes in GM structure were also observed byDriemeyer et al. [24], Scholz et al. [29], andSampaio-Baptista et al. [26,27]. They noted that changes in GM volume can occur as early as after one week of the intervention. These results also confirmed previous reports by Draganski et al. [23] and Boyke et al. [20] showing that the GM volume changes in the visual and parietal areas of the cerebral cortex; however, in some of studies, these changes occurred at later time points [26]. However, most published studies have shown a decrease in GM density at a time point that was evaluated at a longer time after the cessation of the intervention [20,23,24]. However, in the study by Scholz et al. [29], these changes persisted up to four weeks after juggling was stopped. Furthermore, these studies did not reveal a correlation between the final learning effect or daily training amount and structural changes. What is more important, the initial phase of learning correlated with an increase in GM, while refinement of previously learned skills no longer showed a tendency to change the brain structure [24]. This phenomenon was also seen in animal studies, where learning was associated with synaptogenesis and glial hypertrophy, whereas increased motor activity was associated exclusively with angiogenesis – the process of capillary formation [33,34].

Sampaio-Baptista et al. [26] divided participants into a high-intensity exercise group (30 min per day) and a low-intensity group (15 min per day), and they found that the two groups had slightly different changes in brain structure. The group that learned a new skill at a lower intensity was characterized by a decrease in GM volume in the premotor areas and DLPFC, which correlated with task performance, while the high-intensity exercise group was characterized by increased GM volume, which also correlated with performance. In addition, the same authors, in another study, demonstrated that less intensive skill acquisition may rely primarily on previously formed functional connections, increasing their strength while increasing functional connectivity, whereas more intensive practice leads to the formation of new connections, increasing circuit performance [27]. In addition, it was noted that the GM volume at the first measurement (before learning) in the occipito-parietal areas correlated with the learning rate, and also that individuals with higher GM volume in the areas responsible for complex whole-body and bilateral movements maintained the acquired skills for longer [26]. This is because juggling engages, among other areas, the posterior cingulate cortex responsible for efficient shifting of the attention [35,36]. Thus, not only was an increase in GM volume observed after an intervention of learning juggling, but the effect of this learning also depends, to some extent, on the individual’s initial GM level. 

Changes in GM are also observed with other interventions. Müller et al. [15] observed an increase in GM volume in the left precentral bend in a study of seniors after six months of dance instruction. Moreover, other authors, based on observational studies, highlighted the positive effects of long-term dance practice on brain activity, modifying the volume of both the WM and GM in different brain regions [37,38,39]. This rationale should be an incentive to practice and learn new motor skills at every stage of life. Juggling, however, is a specific skill focused on the trajectory of the balls, which implies changes in the areas of the brain mainly responsible for visual motor activities, while requiring the practitioner to constantly maintain an appropriate posture. Therefore, it should mainly improve those used regions that are additionally responsible for the spatial relations of body movements such as reaching or grasping [40] and that are more active during complex two-handed tasks than during unilateral tasks [41].

Observations related to increased WM volume were also observed after the juggling intervention. The results of Scholz et al. [29] showed that there was an increase in FA in the group that undertook juggling compared with the control group. The increase in FA did not correlate with performance in the juggling task, and no correlation was observed between the level of FA prior to the start of training with the skill acquisition speed of the participants.

Three studies included in this review were based on a brief one-day intervention. These studies included, among others, individuals who specialized in juggling tasks. In the study bySchiavone et al. [28], despite the low sample size (*n* = 2), it was noticed that increasing the task’s difficulty enhances the power of neuronal oscillations in all frequency bands, thus extending synchronous brain activity, which may be due to the higher demands of attentional focus and motor control during increasingly difficult motor tasks, even in experienced jugglers. On the other hand, in a second study, Berchicci et al. [21] observed differences occurring in the brains of experienced jugglers and non-jugglers during the performance of a juggling task, which supported the hypotheses of prefrontal neural plasticity during this type of task, due to the fact that they require high levels of coordination, balance, and attention. A study by Carius et al. [22] on young adults engaged in juggling showed that juggling is associated with neurovascular changes in the sensorimotor and visual brain areas. Moreover, they confirmed that greater task complexity modulates these brain regions more. This may be related to the fact that activities that are more difficult are elements that engage the exerciser more cognitively than activities that have already been automated [22]. Additional confirmation of these changes occurring in the brain of experienced jugglers was provided by the study of Gerber et al. [25], where changes were observed to occur specifically in the visual cortex region (V2, hMT+/V5, and IPS) related to motion perception and eye–hand coordination, as well as correlations between GM density (mainly in the hMT+/V5 area) and performance in jugglers. Thus, the development of human brain structures could perhaps be practiced both by motor learning of previously unknown skills and by attempting to learn new motor activities in an already familiar form of physical activity.

It is probable that structural changes in the brain after the juggling intervention may also improve cognitive function among participants who practice this form of activity. This may be supported by the studies of Jansen et al. [42,43] andLehmann et al. [31], who examined the effects of juggling on the outcome of mental rotations among different groups of individuals. The results of all these studies clearly indicated a relationship between motor training in the form of juggling and a shorter reaction time in a mental rotation test, which is associated with improved spatial imagination or mathematical skills. In summary, it can be concluded that learning classical juggling has the potential to develop brain structures.

## 5. Authors’ Conclusions

### 5.1. Limitations

The low number of studies and significant differences in the methodological proceedings prevented the execution of a meta-analysis that could reliably prove the effect of juggling on neuroplasticity. In addition, some of the articles included in the systematic review had a high risk of bias because they were not randomised controlled clinical trials and were therefore based primarily on observations.

### 5.2. Implications for Practice

The effect of physical activity on neuroplasticity is an increasingly widespread topic. Research on this subject has focused on both cyclic endurance forms of physical activity [44] as well as motor learning of novel, sensory-enriched, and often motor control-intensive activities (such as learning to dance) [15,36,45]. The creation of a ball exercise program in the form of juggling, the effects of which on neuroplasticity have been demonstrated in this systematic review, among others, could become one of the ways in which adults can independently attempt to improve their quality of life. Nevertheless, it is important to notice that some of the articles showed, in the follow-up measurements, that an inactivity period tends to reduce the previously obtained positive effects to the same level as before the intervention [20,23,24]. Knowledge of whether a bimanual task in the form of juggling affects the human brain can also contribute to broadening awareness of the impact of various forms of physical activity on neuroplasticity. The advantages of this form of activity, which requires only the use of the upper limbs, are that there is no need for specialized equipment and space, the possibility of safe individual work, and the availability of this form of exercise for people with lower limb aliments. Thus, it can be easily applied in different population groups.

### 5.3. Implications for Research

Future studies with the juggling intervention should meet the criteria for randomised controlled clinical trials. Ideally, a positive control group (where the effect of the intervention results in positive changes in neuroplasticity) and a negative control group (where the effect of the intervention does not result in changes in neuroplasticity) would be used in comparison with the classical juggling intervention. Furthermore, future research should take into account both the participant’s prior juggling experience and learning rate during the intervention to control for subjective task difficulty [26,29]. The results of this systematic review showed that EEG, MRI, fNIRS, and VBM can effectively reveal changes occurring during juggling and after a prolonged intervention with this activity. Moreover, measurements just before the intervention, immediately after the intervention, and some time after the intervention seem to be the optimal number of measurements that can demonstrate both the differences caused by this specific activity and the ability of the observed changes to persist [20,23,24,26,27,29,30]. However, it is very important to note that the brain imaging method should be chosen on the basis of proven protocols that allow reliable comparison of the results obtained with existing ones, and that these are accurately described so that replication of the studies can be performed. Studies of individuals of different ages with a standardized methodology will allow for a robust meta-analysis in the future that may show clearer evidence regarding the effects of juggling on neuroplasticity. It may also be important for scientific research to not only measure structural changes in the brain but also functional changes that affect behavior.

## Figures and Tables

**Table 1:** Summary of participants and assessments.

Study	Country	Participants							Summary of the Intervention Procedure	Assessment
		*n*	Groups	Description	Male	Female	Age (Mean ± SD)	Inclusion and Exclusion Criteria		
Boyke et al. 2008 [20]	Germany	93	Intervention *n* = 25; control *n* = 25; *	Healthy adults	39	54	60.0	Healthy, without dementia, Parkinson’s disease, diabetes, hypertension. None of them could juggle.	Three-month period of juggling training and, after that, a three-month period without juggling.	MRI, VBM
Berchicci et al. 2017 [21]	Italy	28	Expert jugglers (E) *n* = 14; non-jugglers (N) *n* = 14;	Healthy young adults	23	5	E: 32.0 ± 5.9 N: 30.0 ± 5.2	E: able to juggle five or more balls with 10 years of experience. N: No prior experience in juggling. With normal or corrected-to-normal vision; without musculoskeletal injury; no reported history of psychiatric or neurological disease.	Two conditions: N: 1-ball fountain, 2-ball shower E: 2-ball shower, 3-ball shower 20 runs with 15 cycles of throws (150 trials for each task).	Kinematic data, EEG, sEMG
Carius et al. 2016 [22]	Germany	15	No groups	Healthy expert jugglers	15	0	26.3 ± 5.2	Without neurological and psychological diseases. Assessment of expert skills: 5-ball cascade for at least 20 s in eight consecutive trials.	6 trials in different conditions (2 balls in left hand, 2 balls in right hand, 3 balls bimanually, 5 balls bimanually, control for 1 Hz, control for 2 Hz) were performed 8 times for 20 s with a 60 s period of rest.	fNIRS, quantitative rating of juggling expertise
Draganski et al. 2004 [23]	Germany	24	Jugglers *n* = 12; Non-jugglers *n* = 12;	Young adults	3	21	22.0 ± 1.6	No prior experience in juggling.	Three-month period of juggling and, after that, a three months period without juggling.	VBM
Driemeyer et al. 2008 [24]	Germany	20	No groups	Healthy young adults	9	11	26.5	No prior experience in juggling, none suffered from any diseases.	6 weeks of a juggling intervention and, after that, a 6-week period without juggling.	MRI
Gerber et al. 2014 [25]	Germany	32	5-ball-jugglers (5BJ) *n* = 16; controls (C) *n* = 16;	Healthy young adults	28	4	5BJ: 26.9 C: 27.2	Healthy without any psychiatric or neurological diseases.	None	MRI, VBM
Sampaio-Baptista et al. 2014 [26]	United Kingdom	44	High intensity (HI) *n* = 22; low intensity (LI) *n* = 18;	Young adults	22	22	HI: 23.9 ± 3.6 LI: 23.8 ± 3.3	Right-handed with no prior experience in juggling.	HI: 30 min of training per day for 29 days. LI: 15 min of training per day for 29 days. After that, 4 weeks without juggling.	Behavior, MRI, DTI
Sampaio-Baptista et al. 2015 [27]	United Kingdom	64	High intensity (HI) *n* = 22; low intensity (LI) *n* = 18; Controls *n* = 20;	Young adults	33	31	23.8 ± 3.5	Right-handed with no prior experience in juggling	5 days a week of juggling for 6 weeks. After that, a 4-week period without juggling. HI: 30 min of training per day. LI: 15 min of training per day.	Behavior, MRI
Schiavone et al. 2015 [28]	Netherlands	2	Intermediate jugglers (I) and expert jugglers (E)	Intermediate and expert-level jugglers	2	0	40 (I) and 22 (E)	I: able to juggle three balls comfortably for more than 60 s. E: able to juggle five or more balls.	First protocol for I and E: Five conditions (“rest”, “imagery”, “juggle”, “imagery hands”, “no balls”) Second protocol for E: Three conditions (3 balls, 5 balls, 7 balls) repeated three times.	EEG
Scholz et al. 2009 [29]	Germany	48	Intervention *n* = 24; controls *n* = 24;	Healthy adults	26	22	25.02 ± 3.34	Healthy with no prior experience in juggling.	Six-week training period; four-week period without juggling	VBM, MRI
Schultz et al. 2012 [30]	Germany	30	Intervention *n* = 15; controls *n* = 15;	Healthy adults	NI	NI	24.3 ± 3.8	Healthy with no prior experience in juggling.	Two months of juggling until participants were able to juggle a cascade for a minimum of 45 s	MRI

MRI: magnetic resonance imagining, fMRI: functional magnetic resonance imagining; fNIRS: functional near-infrared spectroscopy; DTI: diffusion tensor imagining; VBM: voxel-based morphometry; EEG: electroencephalography; sEMG: surface electromyography; NI: no information; *: others were excluded.

**Table 2:** Summary of study design, period, time points of measurement and outcomes.

Study	Study Design	Period and/or Frequency	Time Points of Measurement	Main Outcomes
Boyke et al. 2008 [20]	FUS	3 months of training	Scan 1—baseline, Scan 2—3 months, Scan 3—6 months	Compared with the first time point (Scan 1), there was an increase in hMT/V5 on the right side during skill performance (Scan 2). This pattern reversed at the third time point (Scan 3). GM volume in the left frontal cortex, cingulate cortex, left hippocampus, and precentral cingulate cortex on the right increased during exercise. After the exercises were discontinued, the change subsided. Transient increases in the GM in hMT/V5, in the hippocampus on the right side, and bilaterally in the nucleus accumbens occurred only in the exercise group. In Scan 3, the effect was reversed.
Berchicci et al. 2017 [21]	RCT	1 session 2 conditions 10 s rest between conditions	During the intervention	The results showed large MRCP, starting before the action of juggling and lasting for the whole duration of the act. The tasks’ difficulty was related to large pN during preparation and execution of the juggling task in both groups. In the more experienced group, the results showed smaller prefrontal and larger frontal activity, mainly during juggling. Juggling practice may induce prefrontal neural plasticity, perhaps because juggling requires important level of coordination, focused attention, and balance during execution.
Carius et al. 2016 [22]	CSS	1 session 6 trials 8 × 20 s 60 s rest between trials	During the intervention	Execution of a complex task such as juggling is related to neurovascular changes in MT/V5 and also to changes in sensorimotor areas (M1, S1, PMC). The complexity of the task seems to modulate the abovementioned brain regions. The 5-ball cascade showed enhanced hemodynamic responses for oxy-Hb when compared with less complex tasks.
Draganski et al. 2004 [23]	RCT	3 months of training	Scan 1—baseline, Scan 2—3 months, Scan 3—6 months	Compared with the first time point (Scan 1), the second time point (Scan 2) showed a bilateral increase in GM volume in the hMT/V5 and in the left posterior medial sulcus. This change decreased at the third time point (Scan 3). These changes occurred only in the training group.
Driemeyer et al. 2008 [24]	FUS	6 weeks of training	Scan 1—Baseline, Scan 2—7 days, Scan 3—14 days, Scan 4—35 days, Scan 5—2 months after training, Scan 6—4 months after training	Compared with the first time point (Scan 1), subsequent time points at which skills were examined (Scans 2–4) showed a bilateral increase in hMT/V5 area, as well as a change in the GM in the frontal lobes, temporal lobes, and the cortex of the cingulate gyrus. This pattern reversed during subsequent time points (Scans 4 and 5).
Gerber et al. 2014 [25]	V-BMS	-	Once/no intervention	Jugglers displayed regional GM density in the occipital and parietal lobes including the secondary visual cortex, the hMT+/V5 area bilaterally and the intraparietal sulcus bilaterally. In jugglers, the results showed a correlation between performance and GM density in the right hMT+/V5 area.
Sampaio-Baptista et al. 2014 [26]	RCT	29 days of training every day (low intensity: 15 min; high intensity: 30 min)	Scan 1—baseline, Scan 2—after training, Scan 3—4 weeks after training	Regions of the brain which had been identified as those where an increase in volume had been observed after juggling training [26] were correlated with the subsequent learning rate in a complex visuo-motor task. In these regions, a significant increase in GM volume after learning was observed in these groups of participants. The results showed that performance outcomes can have an important role in modulating positive structural changes in GM volume over certain time points.
Sampaio-Baptista et al. 2015 [27]	RCT	6 weeks of training, 5 sessions per week (low intensity: 15 min; high intensity: 30 min)	Scan 1—baseline, Scan 2—after training, Scan 3—4 weeks after training	In the low intensity group, the results showed increases in motor network connectivity and decreases in GABA. Scan 3 showed that the increased motor RSN strength was still present. This may suggest that changes in functional connectivity do not require ongoing practice to be maintained. In the high intensity training group, the results showed decreases in connectivity within the motor RSN, and no significant change in GABA. It was shown that lower intensity of practice might rely mostly on previously established functional connections. An increase in the strength of functional connectivity was observed. A higher intensity of practice might cause the formation of new connections and an increase in of circuit efficiency. The phenomenon of decreased functional connectivity was observed.
Schiavone et al. 2015 [28]	CR	1 session, 2 conditions	During the intervention	Higher power of oscillation across the scalp during juggling was observed in the case of expert jugglers. A higher alpha coherence during execution may be associated with hemispheric synchronization in the control and coordination of bimanual tasks. The dominance of the right hemisphere in this case possibly reflects a stronger visuomotor adaptation and a more efficient bimanual motor routine due to extensive practice. Intermediate jugglers were characterized by higher power in the theta and high gamma frequency bands and higher interhemispheric gamma coherence.
Scholz et al. 2009 [29]	RCT	6 weeks	Scan 1—baseline, Scan 2—6 weeks, Scan 3—10 weeks	A significant increase in FA was observed in the WM under the right posterior interparietal sulcus when comparing the first time point (Scan 1) with the second (Scan 2). This change occurred in the training group. After juggling, a significant increase in GM density was observed in the medial occipital and parietal lobe in cortical regions overlying the WM area.
Schultz et al. 2012 [30]	RCT	2 months Progress was reported daily by volunteers	Scan 1—baseline, Scan 2—at the end of intervention period, Scan 3—2 month after end	In juggle group of 225 voxels was obtained within the corpus callosum that showed increased FA between scan 1 and scan 2. This results were obtained just in juggle group. Also a mean of GM density increased in both hemispheres (medial occipital and parietal lobes) from scan 1 to scan 2. This effect was specific for experimental group. Decrease of WM and GM volume was not observed after period without intervention.

FA: fractional anisotropy; GM: gray matter; WM: white matter; GABA: gamma-aminobutyric acid; RSN: resting-state network; PMC: pontine micturition center; M1: primary motor cortex; S1: primary somatosensory cortex; MRCP: movement-related cortical potential; pN: prefrontal negativity; V5: visual cortex area; hMT or MT: middle temporal area; RCT: randomised controlled trial; CR: case report; FUS: follow-up study; CSS: cross sectional study; VB-MS: voxel-based morphometry study.

## Data Availability

Not applicable.
